# Influenza A Virus Entry Inhibitors Targeting the Hemagglutinin

**DOI:** 10.3390/v5010352

**Published:** 2013-01-22

**Authors:** Jie Yang, Minmin Li, Xintian Shen, Shuwen Liu

**Affiliations:** 1 School of Pharmaceutical Sciences, Southern Medical University, Guangzhou 510515, China; E-Mails: yjjy20071030@yahoo.cn (J.Y.); homeostasis@163.com (X.S.); 2 Department of Clinical Laboratory, The First Affiliated Hospital of Jinan University, Guangzhou 510630, China; E-Mail: limm269@126.com (M.L.)

**Keywords:** influenza A virus, hemagglutinin, viral entry, antiviral drugs

## Abstract

Influenza A virus (IAV) has caused seasonal influenza epidemics and influenza pandemics, which resulted in serious threat to public health and socioeconomic impacts. Until now, only 5 drugs belong to two categories are used for prophylaxis and treatment of IAV infection. Hemagglutinin (HA), the envelope glycoprotein of IAV, plays a critical role in viral binding, fusion and entry. Therefore, HA is an attractive target for developing anti‑IAV drugs to block the entry step of IAV infection. Here we reviewed the recent progress in the study of conformational changes of HA during viral fusion process and the development of HA-based IAV entry inhibitors, which may provide a new choice for controlling future influenza pandemics.

## 1. Introduction

Main Influenza A viruses (IAV) cause acute respiratory diseases in humans, birds, and other mammals, representing one of the major threats to public health. Wild birds are the reservoir of influenza A viruses. An avian strain can adapt to the human host and attain human-to-human transmission capability through acquired mutations. An unexpected human adaptation of an influenza subtype or strain rather than currently circulating influenza viruses may cause pandemic flu. The pandemics of 1918 H1N1 (Spanish flu), 1957 H2N2 (Asian flu), 1968 H3N2 (Hong Kong flu) and 2009 H1N1 (swine flu) symbolize the devastating public health and socioeconomic impacts of pandemic flu and keep us alert to any such outbreak. Additionally, seasonal flu is responsible for about 50,000 deaths per year. The H5N1 type IAV, which infected 18 patients in Hong Kong and caused 6 death in 1997, is a potentially serious threat to human health in the near future because of its high mortality (about 60%) and potential human-to-human transmission. 

Influenza viruses mutate frequently because of their segmented RNA genome, making it almost impossible to produce a timely and sufficiently effective vaccine to prevent the potential oseltamivir-resistant H5N1 influenza A viruses epidemic outbreaks. Therefore, it is the only way to use anti-influenza agents for treatment and prevention at the beginning of pandemic outbreak of a virulent influenza strain, which gives time for the development and widespread dissemination of an effective vaccine. There are two classes of anti-influenza drugs up to now available in the clinic, which targeting the M2 ion channel and neuraminidase (NA) expressed on the virus envelope, respectively. Adamantanes block the ion channel formed by the M2 protein, which is critical in the release of viral ribonucleoprotein complexes (vRNPs) into the cytoplasm [1]. Although ion channel inhibitors can be effective against influenza virus infection, they have been reported to cause central nervous system (CNS) side effects. Also, currently circulating IAV strains are mostly resistant to adamantanes [2]. Thus adamantanes are not recommended for a general and uncontrolled use.

Two neuraminidase inhibitors, oseltamivir and zanamivir, were both approved in 1999 for treatment and prevention for acute uncomplicated flu caused by influenza A and B. Neuraminidase inhibitors interfere with the enzymatic activity of the NA protein, which is critical for the efficient release of newly synthesized viruses from infected cells. However, resistant virus strains are constantly emerging, especially to oseltamivir [3]. Different from the oral administration oseltamivir, zanamivir can only be inhaled due to its low bioavailability, which makes the limited use of this drug. In 2009, a new NA inhibitor, peramivir, was authorized for the emergent treatment of certain hospitalized patients with known or suspected 2009 H1N1 influenza.

**Figure 1:**
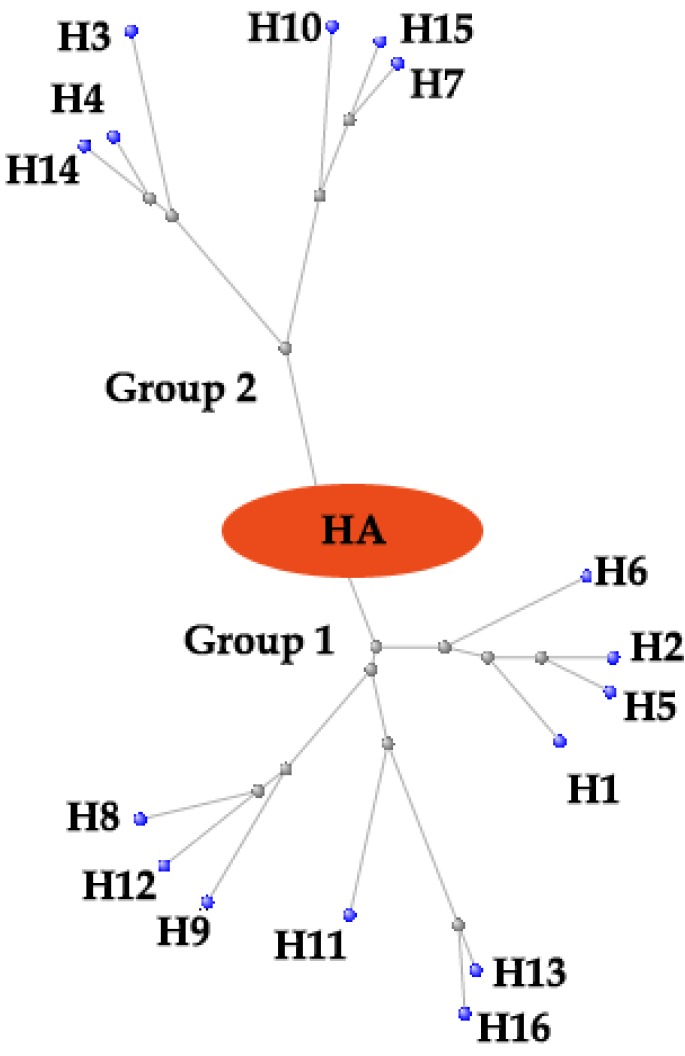
The phylogenetic tree of influenza A virus hemagglutinins (HAs). Constraint-based Multiple Alignment Tool (COBALT) was used and then the figure was revised with Photoshop software. Tree method: Fast Minimum Revolution; Max Seq. Difference: 0.85; Distance: Grinshin General Protein. Grey dots represent nodes; Blue dots represent 16 subtypes of influenza A virus HA proteins.

It seems quite pressing to seek for new anti-influenza medications. Up to now, the life cycle of influenza virus has been well understood, allowing for the validation of several therapeutic targets. Among them, hemagglutinin (HA) is one of the most appealing ones. Till now 16 subtypes of HA have been identified and can be further subdivided into 5 clades and 2 groups (Figure 1). This malleable nature of HA imposes a great difficulty to conduct rational drug design. Furthermore, the variety of HA may be even strengthened by antigenic drift and antigenic shift [4,5]. Here, we described the functional and structural studies leading to the discovery of HA as a new anti-influenza target, and also how structural information is facilitating the rational design of new IAV entry inhibitors targeting HA. 

## 2. The Virus Entry Process and the Function of Hemagglutinin

Hemagglutinin is encoded by the fourth negative-stranded RNA segment of influenza A viral genomes. This RNA segment encodes HA0 with 566 residues. HA0 is post-translationally glycosylated and trimerized with chaperon in endoplasmic reticulum (ER) in infected cell. Subsequently, HA0 undergoes an extra- or intra- cellular cleavage process into HA1 and HA2, which is a critical step for the maturation of influenza virus progenies to acquire their infectivity [6]. Most HA subtypes (H1, H2 and H3) of the circulating influenza viruses in human, have a conserved cleavage site with a sole basic amino acid residue R343 in specified sequence Q/E-X-R, which is only recognized by extracellular tissue-restrict trypsin-like proteases [7]. However, for highly pathogenic avian influenza (HPAI) viruses, the multi-basic HA0 cleavage sites (R-X-R/K-R) of H5- and H7- subtyped HA are recognized by ubiquitously expressed intracellular proteases, facilitating systemic virus spread and greater pathogenicity [8].

**Figure 2:**
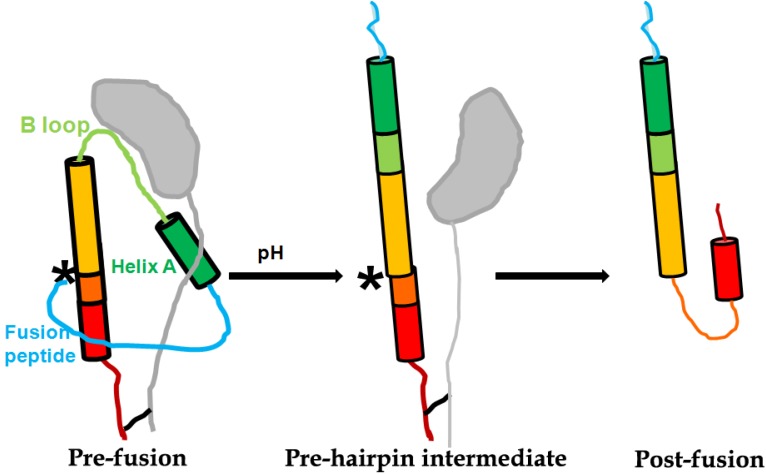
Scheme of pH-induced conformational change of HA2 at the pre-fusion and post-fusion states. The approximate location of HA2 residue 106 is marked with “*”. The figure was adapted from the paper published in J. Virol. [13].

After proper proteolytic cleavage and glycosylation of HA0, disulfide bound subunits HA1 (about 327 residues) and HA2 (about 222 residues) form the fusogenic homotrimer, whose structure resembles a “mushroom” planted in the viral envelope (Figure 2). The globular head of the “mushroom” is mainly constructed with HA1 subdomains, including receptor binding subdomain, vestigial esterase subdomain and antigenic epitope [4]. The receptor binding subdomain of HA1 recognizes α-2,3 or α-2,6 linked terminal sialic acids (SAs) in membrane glycoprotein receptor of host cell, therefore can prime the virus-cell adsorption and endocytosis for virus entry [9]. The stem of the “mushroom” is mainly composed of the inner trimeric HA2 ectodomain subunits, embraced by the N- and C-terminal segments of HA1. The first 23 residues of N-terminal of HA2 is the functional fusion peptide (FP). FP is accommodated in a hydrophobic pocket formed partially by the fusion domain of HA1 [10]. The acidification induced rearrangment of HA1 is a prerequisite for the exposure and release of FP subdomain of HA2 from inner pocket [11]. Subsequently, low pH-triggered HA2 reconformation results in the fusion of the viral envelope with endosome membranes (Figure 2) [12]. The fusion allows the release of viral ribonucleocapsid (vRNP) into cytoplasm of an infected cell, leading to the completion of the entry step.

## 3. Antivirals Targeting HA1

The globular head of HA1, which “crowns” HA2, exposes its outmost surface to the environment, plays an incipient and central role in virus-host interactions, such as binding host receptor, being recognized by host antibodies, evading host immunity system through mutations and glycosylations at specific epitopes. Meanwhile, the electrostatic interactions between positive charged inner surface sites of HA1 and negative charged sites of HA2 stabilize trimeric HA at neutral pH. During fusion, dissociation of HA1 from the trimer spike is necessary for completion of HA2 spring unloading. Being so closely involved in these critical steps in viral entry, HA1 probably contains several critical sites for its interactions with host cell or HA2, which are also potential medicinal targets for developing antiviral drugs. 

### 3.1. Inhibitors Targeting Receptor Binding Sites in HA1

The receptor binding site of HA resembles a shallow pocket, whose structure is composed of β‑barrel motif and α-helices, located on the surface of globular head of the HA1 (residues 116–261). Terminal SAs of cell membrane glycoprotein, the natural receptor for HA-mediated viral adsorption, sit on top of two aromatic residues of HA1 (Y98 and W153 at the bottom of the binding pocket) [14]. Accordingly, a theoretical investigation by molecular dynamics (MD) simulation revealed that Y91 at the bottom of binding pocket of HA1 forms hydrogen bonds with α-2,3 or α-2,6 linked terminal SAs in various HA subtypes [15]. Besides, the binding sites in the pocket are of conserved amino acids that is edged by 190-helix at the top of the pocket, and the 130- and 220-loops located at the front edge and the left side of the pocket, respectively [9]. Above all, the receptor binding sites of HA mainly consist of residues 91, 98, 153 at bottom, 134 to 138 at 130-loop, 183, 190, 194 at 190-helix and 224–228 at 220-loop. Most of these residues are highly conserved among all 16 subtypes of HA. 

The avian influenza viruses HA bind predominantly with α-2,3 linked terminal SAs, therefore they barely overstep the host species barriers to infect human which has only α-2,6 linked terminal SAs in upper respiratory tract and trachea. In other words, to acquire its infectivity in human, avian influenza viruses have to acquire adequate binding avidity to α-2,6 linked terminal SAs through accumulated genetic drift and/or reassortments. A theoretical investigation by MD simulation [15] found that residues in 130-loop (R130, V132, T133, A134, S142), 190-helix (D187) and 220-loop (G222, Q223, A224) of H1-typed HA1 form direct or water-mediated hydrogen bonds with α-2,3 linked terminal SAs. However, to bind H1-typed HA1 with α-2,6 linked terminal SAs, H180 instead of D187 in 190‑helix, G225 instead of G222 in 220-loop of form direct or water-mediated hydrogen bonds. Additionally residue G222 still forms hydrogen bond, but with penultimate galactose of the receptor, rather than the α-2,6 linked terminal SA. Likewise, in H5-typed HA1, residues in 130-loop (S129, V131, S132, S133, S141), 190-helix (H179, N182, E186, K189) and 220-loop (K218, Q223, A224) form direct or water-mediated hydrogen bonds with α-2,3 linked terminal SAs. Compared with interactions between H5-typed HA1 and α-2,3 linked terminal SAs, residues H179, Q223, G224 in H5‑typed HA1 fail to form interactions with α-2,6 linked terminal SAs. 

In association with above reports, several mutations in correspondent sites in 130-loop, 190-helix or 220-loop result in altered HA1 binding specificity for SAs, and therefore may even create a strain of avian influenza A virus transmissible in mammal, such as ferrets [16]. Recent researches indicate two mutations in the avian influenza virus H1N1-typed HA1 (E190D in 190-helix and G225D in 220-loop) change the receptor binding preference from the avian α-2,3 to the human α-2,6 SAs, which resulted in the 1918 H1N1 and 2009 H1N1 virus capable of respiratory transmission between human, and maintaining its lethality and replication [17,18]. However, these two mutations (E190D and G225D) in HPAI H5N1 viruses do not cause a switch in binding specificity [5], though substitutions at positions 129 and 134 in 130-loop of HA1 could change the receptor-binding preference of H5N1 HA from α‑2,3 to both α-2,3 and α-2,6 SAs [19]. Strikingly, in 2012, Imai *et al.* identified four mutations (N158D, N224K, Q226L and T318I) in the H5N1 HA1, which allow this highly pathogenic virus to be transmitted by respiratory droplets between ferrets [16]. 

**Figure 3:**
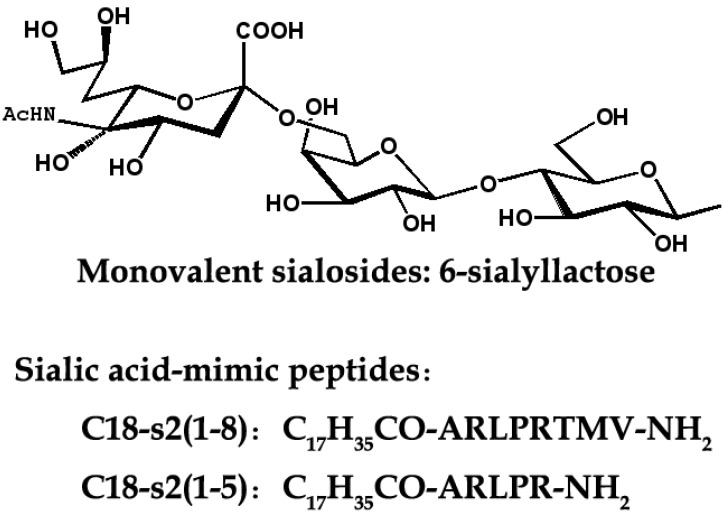
The chemical structures of IAV inhibitors targeting receptor binding sites in HA1.

Based on the fact that SA is HA receptor, SA-based inhibitors can be exploited as potential anti-HA agents. Because one influenza virion usually contains about 350–400 HA trimers on its surface [9], monovalent SA analogs (Figure 3) would be unable to compete with the highly multivalent interactions between viruses and their host cells. Therefore, inhibitors of the bivalent, tetravalent and even polyvalent sialosides afford an enhanced inhibitory activity over monovalent ligands [20]. The polyvalent sialosides show high potency *in vitro* [20]. It is currently acknowledged that recognition of α-2,6 SAs is indispensable for the influenza viruses to gain their efficient transmissibility in human. Therefore, multivalent α-2,6-sialyloligosaccharides could be effective for the protection against newly emerging pandemic influenza virus strains. Otherwise, it is possible to design transition-state α-2,6-SA binding analogues to inhibit receptor-binding and viral adsorption. 

As alternatives to SAs analogs, Teruhiko *et al.* identified peptides (Figure 3) that bind to receptor-binding sites of H1 and H3-typed HAs through a multiple serial selection from phage-displayed random peptide libraries [21]. A docking simulation suggested that the peptides mimic the structure and binding ability of SAs. For example, N-steroyl peptides C_17_H_35_CO-ARLPRTMV-NH_2_ (C18-s2(1-8)) and C_17_H_35_CO-ARLPR-NH2 (C18-s2(1-5)) could inhibit the infection of influenza A/H1N1/Puerto Rico/8/34 virus with IC_50_ values of 3.0 and 1.9 μM, respectively [21]. In 2006, a 20-amino-acid peptide (EB) was found to exhibit broad-spectrum antiviral activity against influenza viruses *in vitro* and *in vivo*, even administered post-infection. Further analysis demonstrated that this EB peptide can specifically bind to the viral HA protein as an entry blocker [22]. In 2009, a smaller peptide P1 (CNDFRSKTC) with similar inhibitory mechanism to EB was also derived [23]. These results indicate that the HA-binding peptides are promising candidates for antiviral drugs as well as anti-HA antibodies.

### 3.2. Inhibitors Targeting Glycosylation Sites of HA1

The HA of IAV often undergoes N-linked glycosylation (NLG) at several sites. NLG of the globular head of HA is known to modulate the antigenicity, fusion activity, virulence, receptor-binding specificity, virus susceptibility to neuraminidase inhibitors, and immune evasion of IAV [24,25]. 

By interfering glycosylation sites, surfactant protein D (SP-D) plays an important role in innate defense against IAV and other pathogens. SP-D (and other collectins including mannose binding lectin (MBL), conglutinin and CL43) inhibits IAV by binding to glycans on the viral HAs. Binding of these collectins to high mannose oligosaccharides on the hemagglutinin (HA) appears to be most important for viral neutralization [26]. Inspired by reported antiviral activity of glycosylation inhibitors, such as nonactin and N-butyldeoxynojirimycin (NB-DNJ) [27,28], it may be possible to achieve an anti‑influenza activity by appropriate glycosylation inhibition strategy. No clear examples of antibodies targeting glycans on HA have yet been reported, suggesting this may be a fertile area for future discovery efforts.

### 3.3. Neutralizing Antibodies Targeting Antigenic Sites in HA1

Analyses of the H1-typed HA sequence variation from natural influenza virus isolates causing epidemics and study of the virus mutations induced by anti-influenza monoclonal antibodies, 5 antigenic sites were identified on H1-typed HA. They were designated as Sa (residues 128–129 near the receptor binding site 130-loop, 156–160, 162–167) and Sb (residues 187–198, overlapping receptor binding site 190-helix), near the top surface of globular head of HA; Ca1 (residues 169–173, 206–208, 238–240) and Ca2 (residues 140–145, 224–225), at subunit interface in the stem region of HA; and Cb (residues 74–79), near the base of the globular head [29]. As for antigenic sites in H3-typed HA, based on study of the HA sequence variation from antigenic variation in the A/H3N3/1968/HK virus, four antigenic sites has been suggested: site A (residues 140–146, near the receptor binding site 130-loop), site B (187–196, overlaps receptor binding site 190-helix), site C (residues 53–54 and 275–278, disulfide bound between Cys52 and Cys277) and site D (residues 201–242, overlay the receptor binding site 220-loop at subunits interface) [30].

By binding antigenic epitopes, antibodies play a central role in the recognition and elimination of invading viruses. Therefore, IAV has evolved low-fidelity polymerases that result in high mutation rates and diverse glycosylations in antigenic sites to escape recognition by neutralizing antibodies [24,31]. Due to viral antigenic shift and drift, most influenza antibodies and vaccines often expire in a short period. Therefore, it is a leading edge to isolate or discover novel broadly neutralizing monoclonal antibodies (bnmAbs) recognizing highly conserved sites. The conserved HA stem domain elicits cross-reactive antibodies, but epitopes in the globular head typically elicit strain-specific responses because of the hyper-variability of this region. However, receptor binding sites are more conserved, since any mutations interfering receptor binding are most likely to impair viral adsorption and infectivity. Based on the fact that some antigenic sites overlap receptor binding sites, these sites are exploited by naturally occurring antibodies in humans. For example, F045-092 neutralizes not only H3N2 but also H1N1, H2N2 and H5N1 viruses. F045-092 shows hemagglutination inhibition activity and do not compete with C179, an antibody thought to bind to the stem region in HA2, but competes with antibodies that recognize sites A and B in HA1, especially residue S136 in 130-loop [32]. Strikingly, another bnmAbs C05 achieve a nanomolar binding with a minimal footprint in the tiny SA binding site in HA1, mainly by inserting just a single heavy-chain complimentarily determining region 3 (CDR3) loop [33]. Similarly, another bnmAbs CH65 also inserts its CDR3 loop into the receptor binding pocket on HA1 [34]. Moreover, receptor-binding bnmAbs F045-092 and CH65 have been isolated from B cells [32,34], suggesting that receptor-binding site antibodies can be found naturally and are potentially inducible by vaccination strategies. 

### 3.4. Host-Targeting Drug Candidates Blocking Viral Adsorption

Though this review talks mainly about the antivirals targeting virus HA, the development of DAS181 still catch our nerves. DAS181 is the only host-targeting drug candidate under clinical development in influenza field. DAS181 is a 46 kDa recombinant fusion protein composed of a sialidase catalytic domain derived from *Actinomyces viscosus* fused with a mucosal cell surface‑anchoring domain [35]. Through cleaving SAs from the host mucosal cell surface, DAS181 inactivates the host cell receptors for viral adsorption, thereby consequently blocks viral entry into respiratory epithelial cells. DAS181 cleaves both the α-2,6-SA and the α-2,3-SA, presents a potent inhibitory effect against a panel of laboratory strains and clinical isolates of influenza A and B viruses, including HPAI strains (H5N1), with *in vitro* EC_50_ values range from 0.04 to 0.9 nM [35]. Recent research revealed that DAS181 is also active against oseltamivir-resisitant H1N1 strains [36]. Besides broad-spectrum antiviral activity, DAS181 treatment may also protect the airway epithelium from inflammation and denudation, which underlies mechanism of preventing secondary bacterial infection [37]. These findings highlight the potential broad spectrum activity of DAS181 against novel and drug-resistant influenza virus strains.

In contrast to DAS181 cleaving receptor SAs from host cell membrane, shielding the receptor SAs on cell membrane is another strategy to interfere the binding event of virus to host cell. Alkylation of two peptides c01 and c03 using N-sterol peptides (C18-peptides), derived C18-c01 and C18-c03 respectively. The alkyl groups of the two peptides are able to promote the formation of peptide assembly that ensures multivalent binding with SA-containing receptors. C18-c01 and C18-c03 respectively could inhibit the infection of influenza A/PR/8/34 virus with IC_50_ values of 3.2 and 6.5 μM [38]. 

## 4. Antivirals Targeting HA2

HA2 subunit, which is linked with virus capsule membrane and owns a highly conservative sequence, conducts the irreversible fusion between viruses and cell endosome membrane at low pH condition and makes the virus genetic material released into the cells. HA2 is constructed by two different length of trend reverse parallel α-helix. There is a B ring formed by two spirals, which connected α-helix and a fusion peptide located in the N-terminal of HA2 with twenty residues (Figure 2). 

### 4.1. Peptide Entry Inhibitors Targeting HA2 Trimer Structure

In the early 1990s, some peptides deriving from the HIV envelope protein gp41 were found to have very strong activity against HIV and to be capable of preventing HIV membrane fusion [39,40]. Among them, a polypeptide, T20, has become the first HIV entry inhibitors targeting gp41 trimer structure with the approval by US FDA [41]. It is a kind of new type of antiviral drugs, suitable for HIV patients with drug resistance. The discovery of HIV peptide enlightened scientists to study type 1 envelope virus entry inhibitors, such as respiratory syncytial virus (RSV), measles virus [42], ebola virus [43], viral encephalitis [44] and severe acute respiratory distress syndrome coronavirus (SARS‑CoV) [45].

Because the influenza virus also belongs to type 1 envelope, scientists have tried to find similar polypeptides like T20 capable of fighting against IAV. But so far there has been no successful report, maybe resulting from the obstacles in the process of developing anti-influenza polypeptides. Unlike HIV, the membrane fusion of influenza virus happened in the endosome with an environment of acidic pH. A polypeptide derived from HA2 subunit cannot pass through the cell membrane to get in the endosome, or become unstable in the acidic conditions, so these peptides cannot effectively prevent membrane fusion mediated by hemagglutinin. A chemical method called hydrocarbon stapling is applied to design spiral polypeptides with the cell membrane penetrability in the study of HIV, with which it was found some peptides have activity to fight against HIV [46,47]. This method may be applicable to search for antiviral polypeptide with the haemagglutinin as the target.

### 4.2. Inhibitors Preventing Low pH-Triggering HA Conformation Change

**Figure 4:**
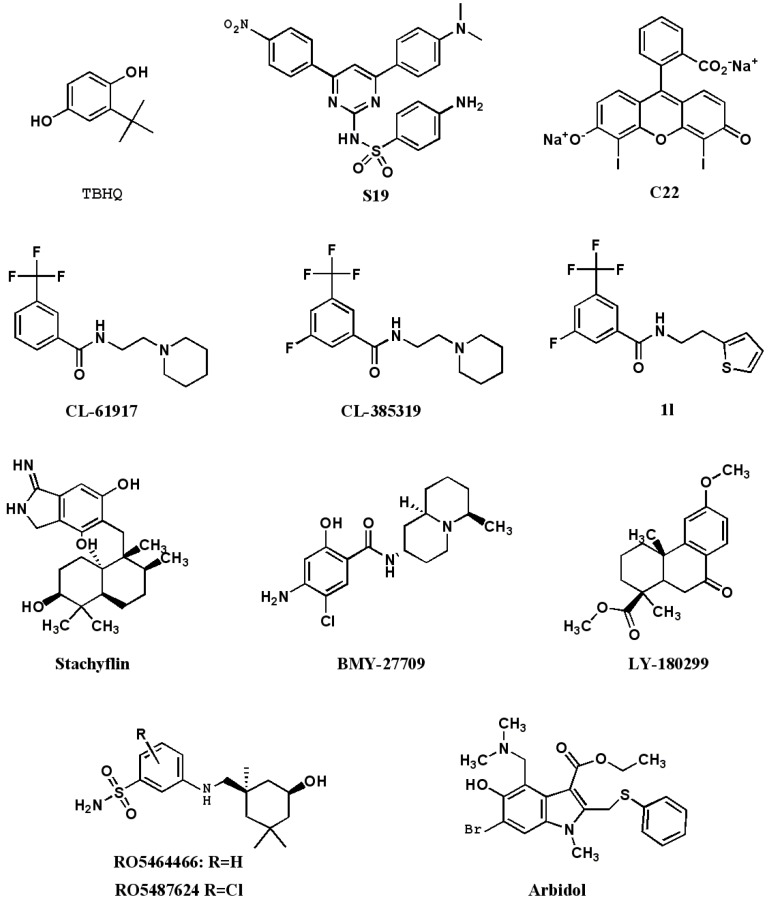
The chemical structures of IAV inhibitors by interfering with the pH-induced conformation changes of HA2.

Another strategy is to find small molecular compounds with the cell membrane penetrability or oral bioavailable compounds which can have interaction with HA to stop the membrane fusion (Figure 4). However, before screening anti-influenza compounds targeting HA2 by high throughput, it is necessary to confirm whether the three polymers possesses the target point suitable for combination before fusion or during the fusion transition state.

The target cavity of HIV gp41 is highly conservative, thus it is not easy to induce resistance for compounds interacted with the cavity, such as T-2544 [48] and ADS-J1 [49], because the resistance mutation of this segment may lead to the loss of HIV virulence. To study of antiviral compounds, it is advisable to devote more efforts to those conservative target points. According to the hemagglutinin, conservative target holes exist in HA2 spiral-screw parts.

In the process of the membrane fusion mediated by HA, after endocytosis into endosome, HA happens irreversible restructuring with the drop of pH. A few small molecular compounds can prevent low pH from triggering HA restructuring, such as benzene anthraquinones and hydroquinone compounds. The most promising benzodiazepine derivative is TBHQ, which can restrain virus infection in the μM concentration [50]. Under the pressure of TBHQ, mutations leading to resistance appear on HA2 subunit which shows that TBHQ is applied to HA2 subunit. The cocrystallization of TBHQ and HA has also been analytic [51]. TBHQ is combined with HA2 subunit, and stabilizes the trimer structure of HA2 under the neutral condition, thus making it disable to restructure in the fusion low pH. Three residues not only owning the competence of ionization but also capable of interacting with TBHQ are the R54 in a HA2 monomer, and E57/E97 in the adjacent HA2 monomer. TBHQ can stop the infection of H14-subtype influenza virus rather than H5N1, which is because in HA2 subunit of H5-subtype hemagglutinin, K58 and E97 form the salt bridge, and E97 on HA2 subunit of H14‑subtype haemagglutinin tends to form the salt bridge with R54. Therefore, K58, existing in HA2 subunit of H14-subtype, can combines with TBHQ. Through the molecular docking screening, two compounds was found with better antiviral activity, S19 (IC50 = 0.8 μM) and C22 (IC50 = 8 μM) [52].

Russia approved an anti-influenza drug, Arbidol, in 1993, which acted by increasing influenza virus HA stability and preventing low pH-induced HA transition to its fusogenic state [53]. However, this potent broad-spectrum antiviral drug is only well-known in Russia and China. A recent study exploiting the characteristics of arbidol-resistant mutants of influenza virus has confirmed its inhibitory mechanism [54]. 

Scientists from Wyeth-Ayerst found three compounds, CL-61917 (N-replaced piperidine), CL‑385319 and CL-62554, can inhibit the activity of H1- and H2- subtype avian influenza, and IC50 reaches a micromole level [55]. When testing the resistant virus strains, it was found that in HA protein sequences the mutation happened on an amino acid located in HA trimer stem area near the HA2 fusion peptide part. By computer-aided drug design the scientists revealed that CL-61917 is able to bind this part of a cavity and may prevent HA restructuring triggered by the low pH, which involves two pathways: (1) Enhance the stability of the residues around the cavity and prevent amino acid protonation caused by acid from damaging interaction force between residues; (2) Stop fusion peptide from moving, and make it disable to form the structure necessary for fusion. CL-385319, the 5-fluoro analog of CL-61917, is more potent than CL-61917 in inhibiting H1 and H2 influenza virus infection. We further found that CL-385319 is also potent in inhibiting H5N1 influenza virus by targeting hemagglutinin on a cavity other than the receptor binding, which at least including the M24 in HA1 and F110 in HA2 [56]. Further mutation study showed V48 in HA2 is also critical for the inhibitory activity of CL-385319 [57]. These critical residues clustered in the stem region of the HA trimer. Extensive computational simulations, including MD simulations, molecular mechanics generalized Born surface area (MM_GBSA) calculations have been carried out to uncover the detailed molecular mechanism. It was found that the recognition and binding of CL-385319 to HA proceeds by a process of ‘‘induced fit’’, whereby the binding pocket is formed during their interaction [57]. Occupation of this pocket by CL-385319 stabilizes the neutral pH structure of hemagglutinin, and thus inhibiting the conformational rearrangements required for membrane fusion. Recently, we further optimized the structure of CL-385319, and found that the 3-fluoro-5-(trifluoromethyl)benzamide segment is critical to the anti-H5N1 virus activity. Substitution the pyrrolidine ring of CL-385319 with thiophene ring can increase the activity to about one fold, making the compound **1l** more attractive candidate for developing as an oral available anti-influenza drug [58].

Researchers from Japan found a few anti-influenza membrane fusion inhibitors, which are stachyﬂin and its derivatives separated from fermentation broth medium of botryoid ear mould [59]. Stachyﬂin can effectively prevent the infection of H1 subtype and H2 subtype avian influenza with IC50 value of a micromole level. Stachyﬂin stops the fusion of virus membrane through the interfering with HA restructuring triggered by low pH. By analyzing the HA gene sequence of stachyﬂin resistant strain, two point mutations was found, K51R and K121E, which are located in HA2 subunit [60]. It suggests that stachyﬂin's binding site is in the HA2 subunit. The same team also designed and synthesized stachyﬂin derivatives, such as acetylstachyﬂin, which have better bioavailability and antiviral activity [61]. But now there is no further report about the progress of this type of antiviral drugs. 

Scientists in Bristol Myers Squibb have found another new kind of fusion inhibitors, BMY-27709, which can inhibit H1- and H2- subtypes influenza virus with IC50 in 3 to 8 μM [62]. Further study found that BMY-27709, combined with HA, prevents low pH triggering HA restructuring. The HA gene sequence analysis of BMY-27709 drug-resistant strain found that the mutation site is located in the N-terminal of HA2 subunit, which showed that compounds prevent HA mediated membrane fusion with an interaction to the N-terminal of HA2 subunit [63].

Eli Lilly researchers found the LY-180299 has a strong inhibition toward A/Kawasaki/86 H1N1 in the early stage of virus replication [64]. After analyzing the HA gene sequence of drug-resistant strain, it was found that the resistant mutations exist on HA1 and HA2 subunit adjacent to interface and the position near the fusion peptide. The pH in which membrane fusion occurs by the drug-resistant strains is 0.3–0.6 units higher than that of the wild type. It suggests that LY-180299 can stabilize the structure of HA neutral state and prevent low pH-triggering HA restructuring.

Tang *et al.* reported a series of benzenesulfonamide derivatives recently with potent anti-influenza activity by targeting HA. These compounds are modified from a salicylamide-based HA inhibitor cis‑2-hydroxy-N-(5-hydroxy-1,3,3-trimethyl cyclohexylmethyl)-benzamide [65]. The lead compound RO5464466 and its 2-chloro analogue RO5487624 can effectively prevent the infection of influenza A/Weiss/43 strain (H1N1) with EC50 values of 210 and 86 nM, respectively. *In vivo* data suggest that RO5487624 has a protective effect on mice that were lethally challenged with influenza H1N1 virus [66]. Mechanism of action studies indicated that these compounds inhibit the virus membrane fusion with host endosome membrane by binding to HA and stabilizing the prefusion HA structure. These compounds also have significantly improved metabolic stability which could be developed as the first generation of orally bioavailable HA inhibitors.

Chloroquine is an inexpensive and widely available anti-malaria drug. Recently, *in vitro* assays suggest that chloroquine may have utility in the treatment of several viral infections including influenza. Vigerust *et al.* tested the *in vivo* effectiveness in ferret models. The results showed that clinical signs and viral replication in the nose of ferrets were not altered by treatment [66]. Just recently, a finished randomised, double-blind, placebo controlled trial showed that chloroquine does not prevent infection with influenza [67]. More cases in the chloroquine group are found catching laboratory-confirmed influenza infection, although generally well tolerated by a healthy community population. This dampens the enthusiasm for potential utility of the drug for humans with influenza.

Most of the founded small molecules are more potent in inhibiting group 1 IAV than group 2 IAVs. Vanderlinden *et al.* reported a new series of N-(1-thia-4-azaspiro[4.5]decan-4-yl)carboxamide inhibitors which show marked antiviral activity against group 2 influenza A/H3N2 viruses [68]. The lead compound 4c has an IC50 of 9.6 μM and a selectivity index of 10. This compound has specific activity against the H3 subtype, but not the H1, H5 and H7 subtypes.

### 4.3. Anti-HA2 Monoclonal Antibodies

Among all structural proteins of influenza viruses, hemagglutinin plays an important role in viral binding to receptors, virus entry and membrane fusion. Antibodies specific for HA have been shown to inhibit and/or neutralize virus infection in vaccinated hosts and anti-HA monoclonal antibodies (mAbs) are regarded as one of the important passive therapeutics for treatment of influenza. There are some companies, such as Theraclone, intend to screen human donors for broadly neutralizing, cross‑reactive human antibodies to hemagglutinin. Current flu vaccines mainly provoke antibodies that target the head region of HA, but that part of this protein changes easily. Therefore, mABs recognizing a more conserved and efficacious epitope may broadly neutralize homologous and heterologous strains with different clades of IAV infection and prevent formation of escape mutants. 

Although antibodies induced by HA2 do not have virus-neutralizing activity, monoclonal antibodies (mAbs) specific to the fusion peptide were able to inhibit the HA fusion activity and to reduce virus replication *in vitro*, and also *in vivo* [69,70]. Antibody CR6261 was first isolated from a person who had been vaccinated against seasonal flu [71]. Virus neutralization test showed CR6261 exhibits broad neutralizing activity against antigenically diverse H1-, H2-, H5-, H6-, H8- and H9-typed group 1 influenza viruses. More recent tests have shown that CR6261 also combats the novel H1N1 virus that caused the 2009 pandemic. Moreover, CR6261 strongly outperformed oseltamivir for influenza prevention and treatment in a preclinical study. Researches revealed that the Crucell’s mAb CR6261 binds to a part of the H surface protein that does not change when the virus mutates—which it does with great ease. The H surface protein is crucial for replication of the influenza virus. Once this site is bound by an antibody, the virus cannot fuse with membranes inside the host’s cells and therefore cannot reproduce. This mAb neutralized the virus by stabilizing the prefusion state and strongly inhibiting the conformational change of HA at the fusogenic pH. CR6261 cannot neutralize the H3‑subtype likely due to glycosylation at residue position of N38. It was also undesired that the conserved epitope recognized by CR6261 was not particularly exposed in intact virus. The CR6261 epitope identified here should accelerate the design and implementation of improved vaccines that can elicit CR6261-like antibodies, as well as antibody-based therapies for the treatment of influenza [72]. Besides CR6261, new broadly neutralizing mAbs F10 and FI6 are also bind to a common conserved region of HA [73].

Monoclonal antibodies that target highly conserved viral epitopes might offer an alternative protection paradigm. Anti-M2e mAbs TCN-032 is an IgG monoclonal antibody which provide a broadly efficient protection for the treatment of influenza A virus. TCN-032 can binds to a novel conformational epitope on the N-terminus of the M2e protein [74]. The epitope recognized by TCN‑032 is conserved in over 98% of known influenza A strains, including avian and swine strains. TCN-032 also has demonstrated potent protection *in vivo* against H5N1 and 2009 S-OIV H1N1 influenza strains. Clinical trials are undergoing to show its safety and effectiveness. Besides, broadly neutralizing, cross-reactive human antibodies to hemagglutinin are screening from human donors. To date, several candidates have been identified that bind and broadly neutralize Group 1 as well as the H3 subtype of Group 2 influenza strains [75].

## 5. Inhibitors Targeting the Cleavage of the HA0 to HA1 and HA2

As the H3-subtype avian flu virus X31 HA crystal structure and H1, H5, H9, H7-subtype avian influenza virus HA crystal structure are unfolded, people get a better understanding of haemagglutinin. The crystal structure show that HA is trimer forms. Each monomer of HA can be hydrolyzed into subunits HA1 and HA2 by host protease. The two hydrolyzed subunits are then connected by disulfide (Figure 2). The structure of HA precursor (HA0) and the structure of the hydrolysis are very alike. Only after six residues of the C-terminal of HA1 and 12 residues of the N-terminal of HA2 subunit distributing can they fuse. Especially the 12 residues of HA2 cover polar amino acids in HA0 which is exposed to liquid environment. This makes the HA vulnerable to hydrogen ion excitation and its structure changes [76]. 

**Figure 5:**
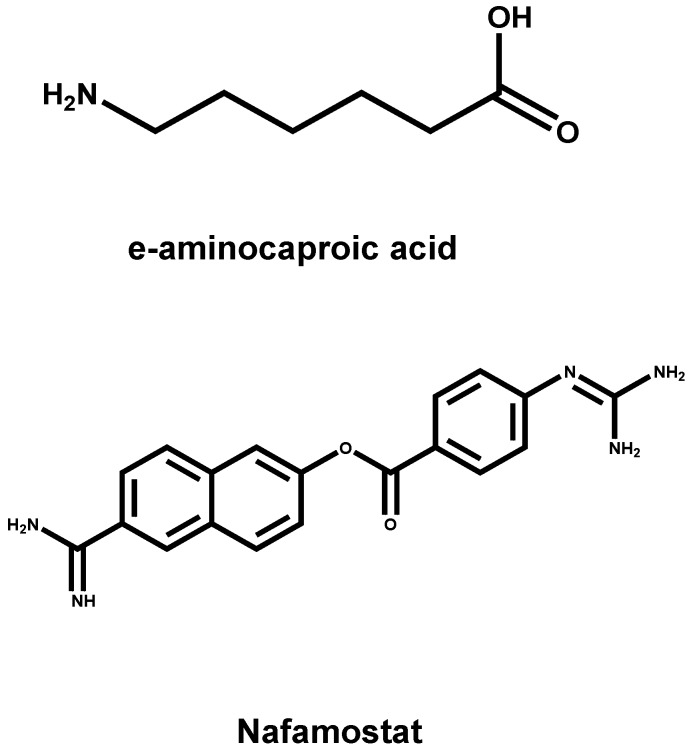
The chemical structures of IAV inhibitors by suppressing the cleavage of the HA precursor HA0 into the functional HA1 and HA2 subunits.

The hydrolysis of HA0 into HA1 and HA2 is a necessary step for avian influenza virus to be contagious. Therefore, if any molecule can prevent HA0 from being hydrolyzed into the subunits of HA1 and HA2, it possesses the ability to fight against viral infection. Some serine protease inhibitors, such as anti-protease peptide constituted of 58 amino acids [77], e-aminocaproic acid [7], Nafamostat [78], pulmonary surfactant (a kind of surface active lipoprotein complex) [79] and mucous protease inhibitors [80], can reduce the hydrolysis of HA0 and the infection of influenza viruses in both cell models and animal models (Figure 5). As a kind of injections to treat the symptoms of bleeding, cow pancreas inhibitor, protease peptide (Trasylol bayer), has been applied to the clinical practice. However, it was withdrawn from the clinical application with the study finding that the drug could increase mortality in 2008 [81]. 

There are some FDA approval pulmonary surfactants, such as Exosurf, Curosurf, Infasurf and Survanta, all of which can enhance lung compliance, and prevent the baby respiratory distress syndrome. Quasi peptide inhibitor (dec-RVKR-CMK) resisting alkaline amino acid protease can inhibit the replication of H7 avian flu with high pathogenicity [82]. Recently, some researchers reported the MPSL/TMPRSS13 can hydrolyze H5 and H7 subtypes [83]. Therefore, natural inhibitors in the human body against MPSL/TMPRSS13 can serve as a lead compound to develop antiviral drugs which targeting haemagglutinin. It was found that the quasi peptide inhibitor also can restrain MPSL/TMPRSS13 and other insulin proteases such as plasmin, which may be the mechanism of action for this inhibitory peptide against influenza virus infection.

## 6. HA-Targeting Natural Products

**Figure 6:**
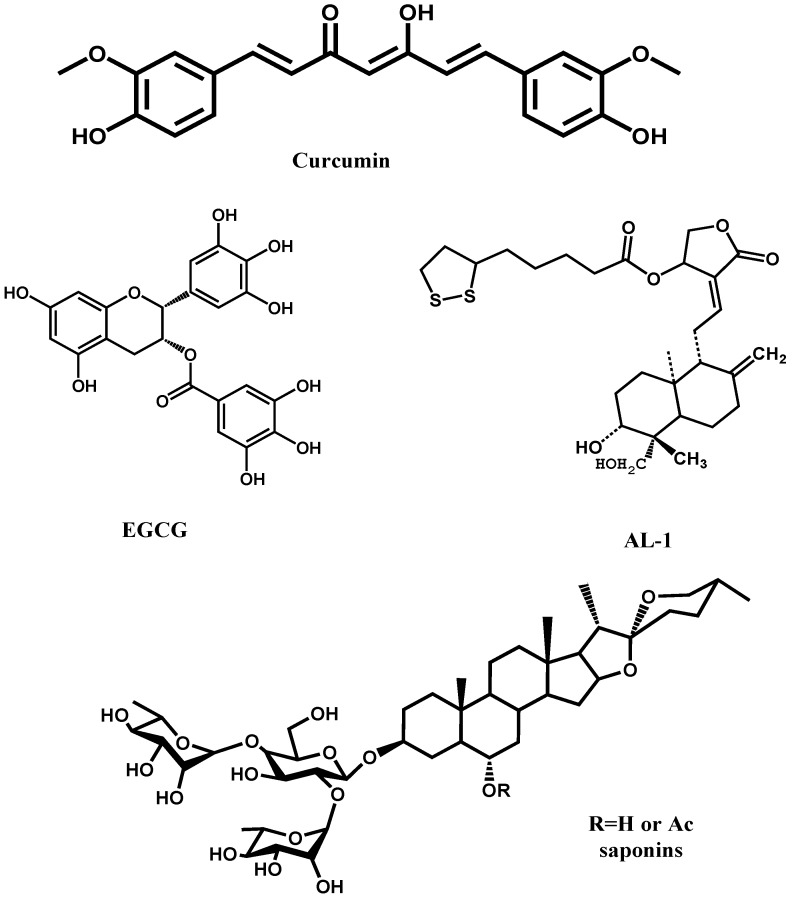
The chemical structures of IAV inhibitors targeting HA1 to block virus binding to receptor.

In addition to above inhibitors, some natural molecules also possess good inhibitory activity against influenza A virus infection (Figure 6). For example, catechins isolated from green tea, like EGCG, were found to exhibit mild anti-influenza effect [84]. Further modifications of catechins derived a set of better inhibitors. To be better anti-influenza drug candidates, catechin derivatives possess broad spectrum anti-influenza activity. Besides, curcumin, the widely used spice and coloring agent in Indian food, was proved to be a good virus entry inhibitor targeting HA with EC_50_ value of 0.47 μM [85]. Modification of curcumin may produce a series of novel HA targeting inhibitors. Another kind of small molecule inhibitor is derived from andrographolide, like AL-1. AL-1 showed significant activity against avian influenza A (H9N2 and H5N1) and human influenza A H1N1 viruses *in vitro* [86]. AL-1 is capable of direct interfering with viral HA to block viral binding to cellular receptors as was demonstrated by its inhibitory activity on viral adsorption to red blood cells. In 2009, Li’s group in China reported the first three small saponins molecule which inhibit HA. These three compounds can potently inhibit the entry of a H5N1 virus (A/Viet Nam/1203/2004) with IC_50_at low μM level. Further modifications revealed that the reduction of R1 to a disaccharide chain would abolish the inhibitory activity of these inhibitors [87]. Therefore, anti-influenza agents from natural products, especially those from Traditional Chinese Medicine (TCM), are promising lead compounds. Some lead compounds from TCM are extensively and intensively reviewed by Xu *et al.* in 2010 [88].

## 7. Conclusions

HA, the major surface protein of IAV, mediates the viral binding, membrane fusion and viral entry. Determination of the crystal structures of HA0 provides important information for understanding the pH-induced conformational changes of HA at the pre-fusion, intermediate and post-fusion states for development of anti-influenza drugs. Although a series of anti-influenza drugs targeting the NA and M2 ion channel are currently available, the emergency of drug-resistance viruses has raised the great concern on their ineffectiveness against the newly emerging IAVs and the HPAI viruses. Thus, it is essential to develop novel anti-IAV drugs with new targets. A number of protein-based or small molecule anti-IAV agents have been shown to interfere with the HA-mediated membrane fusion by targeting the receptor binding, blocking the cleavage of HA0, or by inhibiting the low pH-mediated conformation changes of HA. It seems not easy to find or design small compounds targeting the binding event of influenza virus, but the conformational change of HA2 which mediates membrane fusion, is a promising target for developing anti-influenza drugs. Novel influenza virus entry inhibitors may provide more selections for combination therapy with NA inhibitors and M2 ion channel blockers for treating and preventing influenza virus infection and potential pandemic outbreak.
